# Characterization of Antibacterial Cell-Free Supernatant from Oral Care Probiotic *Weissella cibaria*, CMU

**DOI:** 10.3390/molecules23081984

**Published:** 2018-08-09

**Authors:** Hae-Soon Lim, Ji-Eun Yeu, Sang-Phil Hong, Mi-Sun Kang

**Affiliations:** 1 Department of Dental Education, School of Dentistry, Chonnam National University, 77 Yongbong-ro, Buk-gu, Gwangju 61186, Korea; hs1964@jnu.ac.kr; 2 Research Institute, Oradentics Inc., 1805-ho, 25 Seongsuil-ro-4-gil, Seongdong-gu, Seoul 04781, Korea; ji-eun85@oradentics.com; 3 Division of Strategic Food Research, Korea Food Research Institute (KFRI), 245, Nongsaengmyeong-ro, Iseo-myeon, Wanju-gun, Jeollabuk-do 55365, Korea; sphong@kfri.re.kr

**Keywords:** *Weissella cibaria*, antibacterial, cell-free supernatant, organic acid, secretome, probiotic

## Abstract

Recently, studies have explored the use of probiotics like the *Weissella cibaria* strain, CMU (oraCMU), for use as preventive dental medicine instead of chemical oral care methods. The present study was conducted to investigate the antibacterial properties of the cell-free supernatant (CFS) from this bacterium. Cell morphology using the scanning electron microscope, and the antibacterial effect of CFS under various growth conditions were evaluated. The production of hydrogen peroxide, organic acids, fatty acids, and secretory proteins was also studied. Most of the antibacterial effects of oraCMU against periodontal pathogens were found to be acid- and hydrogen peroxide-dose-dependent effects. Lactic acid, acetic acid, and citric acid were the most common organic acids. Among the 37 fatty acids, only 0.02% of oleic acid (C18:1n-9, *cis*) was detected. Proteomic analysis of the oraCMU secretome identified a total of 19 secreted proteins, including *N*-acetylmuramidase. This protein may be a potential anti-microbial agent effective against *Porphyromonas gingivalis*.

## 1. Introduction

Oral health can have a great impact on our general health and quality of life. Several oral care products are commonly and widely used for oral hygiene. However, the use of several antibacterial agent-containing oral care products may disrupt the natural microbial ecology of the mouth. This could lead to an overgrowth of opportunistic pathogens or exogenous microorganisms. Recently, the potential application of probiotics for oral health has been reported by many researchers [1,2,3]. Oral care probiotics have been commercialized as substitutes for the conventional treatment for oral diseases, including halitosis, dental caries, gingivitis, and periodontitis [4,5,6,7,8].

Probiotics are live organisms that confer health benefits to the host when administered in adequate amounts [9]. They produce functionally valuable substances, such as exopolysaccharides, folate, and antioxidants [10]. They also fight pathogens by altering the surrounding environment through production of antibacterial compounds and by competition for space and nutrients [11], and they modulate immune and inflammatory responses as well [12]. While several strains of lactobacilli 41 are known to have probiotic effects against oral pathogens, *Weissella cibaria* has largely been ignored.

A lactic acid bacterium, *W. cibaria* can be found in a multitude of places—not only in human saliva, but also in traditional fermented foods, such as kimchi, tarhana, and sourdough [13,14,15,16]. *W. cibaria* CMU, CMS1, CMS2, and CMS3 were isolated from the saliva of healthy infants that were caries-free [13] and patented as strains to suppress halitosis (US7250162B2). *W. cibaria* CMU (oraCMU) was recently commercialized as an oral care probiotic in South Korea owing to its beneficial effect on oral cavities [4]. Since the early 2000s, our research group has been studying the effects of *W. cibaria* on oral health through clinical studies [17,18]. Our previous studies have shown that *W. cibaria* can function in several ways: By directly inhibiting the growth of harmful bacteria [17] through competitive adhesion to the oral cavity [19]; through immune-regulatory actions to prevent inflammatory cytokines [20]; and by inhibiting *Streptococcus mutans* biofilm formation by converting water-insoluble glucans into water-soluble ones [18]. *W. cibaria* has been known to produce antibacterial substances, such as hydrogen peroxide, that can inhibit the proliferation of malodor-inducing bacteria [17]. However, it is unknown whether *W. cibaria* can produce other compounds that can act against periodontopathogens, and whether the notable bactericidal properties are due to pH changes or secretory components.

Therefore, the aim of this study was to identify the antibacterial component of the cell-free supernatant (CFS) from oraCMU. Our investigation was carried out with a focus on estimating hydrogen peroxide production capabilities and identifying organic and fatty acids through high-performance liquid chromatography (HPLC) and gas chromatography (GC). Furthermore, the potential secretory proteins responsible for the antibacterial activity were analyzed using 2D gel electrophoresis, as well as matrix-assisted laser desorption/ionization time-of-flight mass spectrometry (MALDI-TOF MS) analysis.

## 2. Results

### 2.1. Cell Morphology of oraCMU

**Figure 1:**
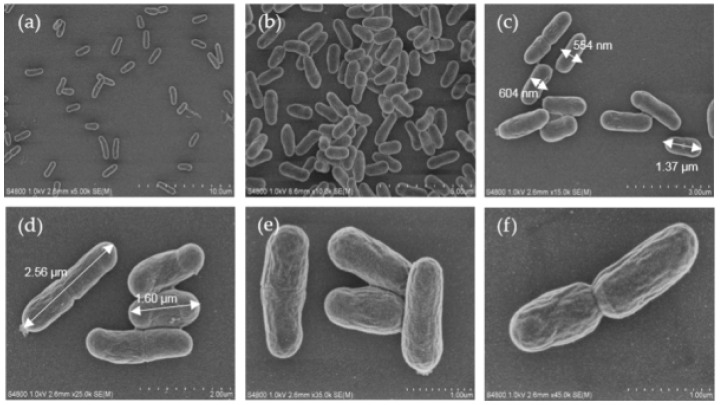
Scanning electron microscopic (SEM) images of oraCMU at various magnifications: 5000× (**a**), 10,000× (**b**), 15,000× (**c**), 25,000× (**d**), 35,000× (**e**), and 45,000× (**f**).

In order to observe the cell morphology of oraCMU, scanning electron microscopic (SEM) analysis was used at various magnifications, such as 5000×, 10,000×, 15,000×, 25,000×, 35,000×, and 45,000×. The bacteria were grown in MRS broth for 16 h, as shown in Figure 1. Cells were observed as short rods growing in pairs and were 0.5–0.7 µm wide and 1.3–2.5 µm long.

### 2.2. Characterization of Antibacterial Substances

**Figure 2:**
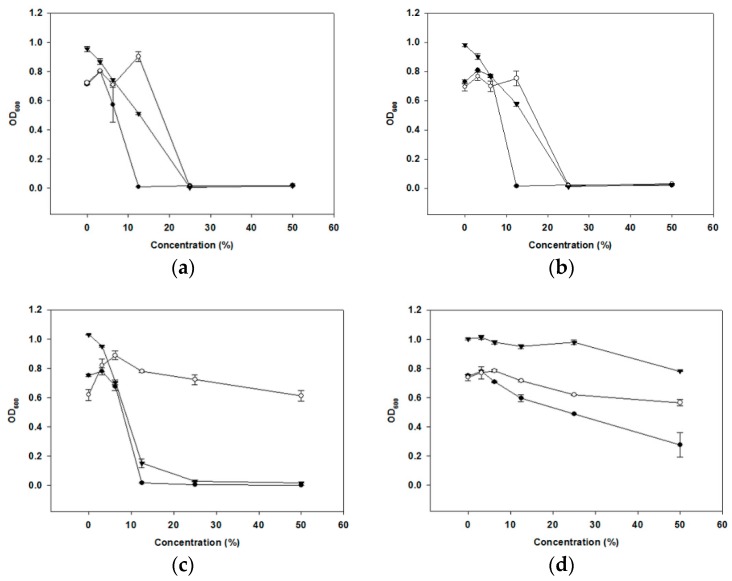
Dose-dependent effects of organic acid, hydrogen peroxide (H_2_O_2_), and a bacteriocin-like compound (BLC) in the cell-free supernatants (CFS) of oraCMU against periodontopathogenic bacteria. Optical density at 600 nm (OD_600_) of the cell suspension was measured. (**a**) Untreated CFS effect; (**b**) organic acid-dependent effect was measured using proteinase K and catalase-treated CFS; (**c**) H_2_O_2_-dependent effect was measured by the neutralized and proteinase K-treated CFS; (**d**) BLC-dependent effect was evaluated by the neutralized and catalase-treated CFS. *P. gingivalis* (●); *Fusobacterium nucleatum* (○); *P. intermedia* (▼).

The CFS was treated in different ways to find out the components of cell-free supernatants (CFS) (organic acids, hydrogen peroxide (H_2_O_2_) or bacteriocin-like compounds (BLC)) that may be responsible for the anti-periodontopathogenic activity of oraCMU. As shown in Figure 2, the untreated CFS inhibited the growth of all periodontopathogens in a dose-dependent manner. The inhibitory effect of oraCMU was not affected by proteinase K and catalase treatment (Figure 2b). The H_2_O_2_-dependent effect of CFS was found to act against *Porphyromonas gingivalis* and *Prevotella intermedia*. On the other hand, BLC-dependent antimicrobial action of CFS was effective only against *P. gingivalis*. However, most of the antibacterial activity of CFS disappeared after neutralization.

### 2.3. Kinetics of H_2_O_2_ Production

**Figure 3:**
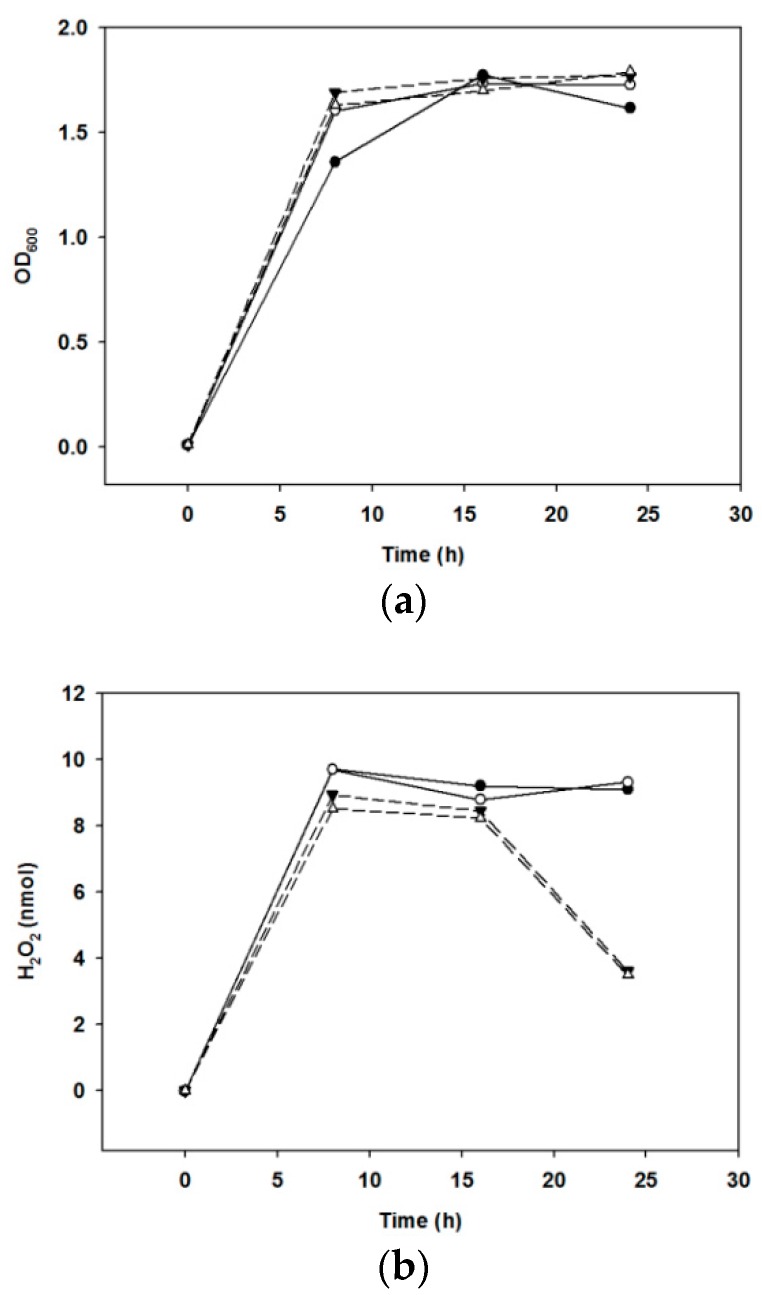
Growth and H_2_O_2_ production under various conditions. (**a**) Growth was monitored spectrophotometrically at 600 nm. (**b**) Production of H_2_O_2_, determined as mentioned in Section 4. Solid lines, aerobic incubation at 30 °C (●) and at 37 °C (○); broken lines, anaerobic incubation at 30 °C (▼) and at 37 °C (△).

The amount of H_2_O_2_ produced by the growth of bacteria was compared when cultivated under various culture conditions. We found that the optical density at 600 nm increased under anaerobic culture conditions until 24 h, but the production of H_2_O_2_ decreased (Figure 3).

### 2.4. Determination of Organic and Fatty Acid Production

We examined the presence and concentration of five different organic acids (viz. oxalic acid, citric acid, succinic acid, lactic acid, and acetic acid) in the CFS by using the HPLC technique. The concentration of lactic acid was found to be highest at 6.42 mg/mL, followed by acetic acid and citric acid, which showed concentrations of 2.78 mg/mL and 0.88 mg/mL, respectively. Oxalic acid and succinic acid were not detected. When the presence and concentration of 37 fatty acids in the CFS were examined by GC, only oleic acid (C18:1n-9, *cis*) and palmitic acid (C16:0) were detected in the CFS, while the other fatty acids could not be detected (Table 1).

### 2.5. Secretome Analysis

**Figure 4:**
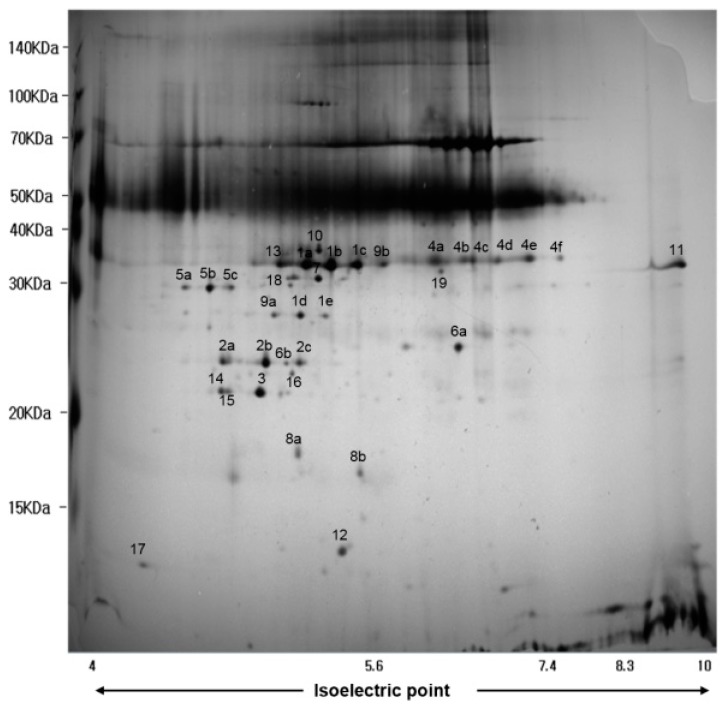
2D-gel electrophoresis of secreted proteins in the CFS of oraCMU after 16 h incubation at 37 °C.

In order to identify secreted proteins in the CFS of oraCMU, proteins were separated by 2D gel electrophoresis (Figure 4). Using MALDI-TOF MS, a total of 19 secreted proteins were identified (Table 2). Amongst the identified proteins, the high-intensity spot proteins found were as follows: type 1 glyceraldehyde-3-phosphate dehydrogenase; *N*-acetylmuramidase; TIGR00266 family protein; leukocyte elastase inhibitor; l-lactate dehydrogenase; and a hypothetical protein. Spot numbers 1, 5, 9, 10, 16, and 19 were involved in energy production and conversion; spot number 2 in the hydrolysis of the peptidoglycan layer; spot numbers 3 and 6 were of unknown function; spot number 4 was a leukocyte elastase inhibitor; spot number 7 was involved in NADPH-dependent reductase; spot number 8 was related to DNA recombination and repair; spot number 11 in the outer membrane; spot numbers 12, 13, and 14 in nucleotide metabolism; and spot numbers 15, 17, and 18 in carbohydrate *trans*port and metabolism.

## 3. Discussion

Although a number of treatment approaches aim to inhibit the growth of pathogenic bacteria, probiotics have been applied as a novel way for the improvement of oral health [1,2,3]. They have been used to replace conventional antibiotic therapy, which has been increasingly ineffective due to increased bacterial resistance [4,5,6,7,8]. Probiotics not only have antimicrobial activity, but also inhibit the reappearance of oral pathogenic bacteria. In order to choose the best oral care probiotics, it is important to select bacteria that originate from the oral cavity which can withstand poor oral conditions, have low acid production, have antimicrobial activity, and are capable of inhibiting biofilm formation. The most common probiotic bacteria belong to the *Lactobacillus* species. Contrary to conventional belief, most lactobacilli are highly acidic and not suitable as oral care probiotics, as they have the potential to cause tooth decay. Certain strains of *W. cibaria* have been studied as optimal oral care probiotics [4,13,17,18]. Previous studies have shown that oraCMU strains produce less acid and more hydrogen peroxide than other commercial bacteria, and thereby can prevent halitosis or dental caries [4].

The surface morphology of the dextran of *W. cibaria*, examined using SEM, has been reported [21]. However, as far as we are aware, this is the first report to analyze the morphology of *W. cibaria* using SEM. In this study, the cells of *W. cibaria* were found to be short rods, 0.5–0.6 µm wide and 1.2–2.5 µm long (Figure 1). This result is consistent with the known fact that the cells of *W. cibaria* are short rods growing in pairs. However, the size determined was slightly different from the previously reported sizes of them being 0.8–1.2 µm in width and 1.5–2.0 µm in length [22].

Dental plaques are complex biofilms that accumulate on tooth enamel surfaces. Among over 700 bacterial species that form biofilms in the oral cavity, pioneer bacteria which initiate the colonization of the enamel salivary pellicle in a continuous fashion, followed by secondary bacterial colonization. Oral streptococci are the predominant species in the initial colonization stage. They produce antibacterial substances, such as H_2_O_2_, as a byproduct of aerobic metabolism. H_2_O_2_ acts as a protective mechanism for the initial colonizers against competing species [23], and is one of the typical antimicrobial substances produced by lactic acid bacteria (LAB) [10]. Previous studies have shown that oraCMU has the best ability to produce H_2_O_2_ than other commercial oral care probiotics [4]. A high degree of H_2_O_2_ could therefore be sufficient to inhibit the growth of bad breath-causing bacteria [18]. In the present study, oraCMU showed H_2_O_2_–dependent inhibition of the periodontopathogenic bacteria *P. gingivalis* and *P. intermedia* (Figure 2).

Hydroxyl radicals react with nucleic acids to damage genes, increase membrane permeability, limit membrane *trans*port, and denature proteins within cells [24]. H_2_O_2_ is a strong antimicrobial substance produced by oraCMU. We aimed to determine whether H_2_O_2_ production was highest when oraCMU was cultivated under optimal conditions. In our present study, although no difference was observed in the growth of oraCMU under aerobic or anaerobic conditions, the H_2_O_2_ production under anaerobic conditions after 16 h decreased faster than when under aerobic conditions. This result is similar to a previous report that showed a higher H_2_O_2_ secretion by lactobacilli under aerobic conditions than under anaerobic conditions [25].

A large number of LAB inhibits the growth of bacterial pathogens by producing metabolites, such as acetic acid and lactic acid, and by lowering the pH [10]. In the present study, the antibacterial activity of CFS from oraCMU was not lost after a catalase and proteinase K treatment. Additionally, CFS, whose pH was adjusted to 7 and treated with proteinase K, still showed antibacterial activity, except on *F. nucleatum.* This suggests that the antibacterial activity of oraCMU might principally be attributed to some acids and H_2_O_2_.

In this study, HPLC analysis confirmed the presence of lactic, acetic, and citric acids in the CFS. The growth of *P. gingivalis*, *P. intermedia*, and *F. nucleatum* strains were also inhibited in the presence of lactic acid, acetic acid, and citric acid. The inhibitory effect of these acids has been reported to occur due to diffusion across the cell membrane towards the more alkaline cytosol, which interferes with the essential metabolic functions of the cell. The toxic effects of these acids include a decrease in intracellular pH and dissipation of membrane potential [26].

Oleic acid has been reported to be bactericidal against group A streptococci [27]. Wong et al. [28] also identified oleic acid to be the major component of the lipid fraction in the CFS of LAB strains that exhibited a strong inhibitory effect against *Staphylococcus aureus*. Nuñez de Kairuz et al. [29] reported that the H_2_O_2_-generating system was specifically induced by one of the saturated fatty acids from 4:0 to 16:0, or oleic acid. The bacterial membranes of lactobacilli typically consist of linear saturated, unsaturated, and cyclopropane fatty acids [29]. Oleic acid was incorporated into the membranes of lactobacilli when grown in an MRS broth supplemented with Tween 80, which consists of up to 90% oleic acid. It has been reported that only oleic acid protected *L. rhamnosus* GG when exposed to an acidic environment [30]. In the present study, we used GC to identify the presence of potential anti-microbial fatty acids in the CFS, and our results identified only oleic acid in the CFS, suggesting that oleic acid from the cell wall components might contribute significantly to the antibacterial activity of oraCMU.

The present study showed that there was a significant decrease in antibacterial activity in the neutralized and catalase-treated CFS. However, oraCMU still showed growth inhibition against *P. gingivalis* in this condition. This suggests the presence of another antimicrobial substance, such as BLC. To date, there have been no reports regarding the proteins secreted by *W. cibaria*, meaning that this is the first paper to report on the proteomic analysis of secretory proteins of the *W. cibaria* strain. In our study, type 1 glyceraldehyde-3-phosphate dehydrogenase (GAPDH) was detected most frequently among the identified proteins. GAPDH is known to be an essential enzyme involved in the production and conversion of intracellular energy, and is found not only in bacteria but also in fungi and eukaryotes [31]. These cytoplasmic enzymes are commonly known as housekeeping molecules. Recently, it was also reported that cell wall-associated GAPDH, found on the surface of non-pathogenic lactobacilli, was involved in the adherence [32].

In addition to GAPDH, other proteins which are known to be involved in energy production and conversion, such as l-lactate dehydrogenase, NAD-dependent GAPDH, *N*-acetylglucosamine-6-phosphate deacetylase, 2,3-diphosphoglycerate-dependent phosphoglycerate mutase, and zinc-dependent alcohol dehydrogenase, were identified. In the present study, several other proteins were also identified, including the BMP family *trans*porter substrate-binding proteins in the outer membrane, enzymes involved in nucleotide metabolism, proteins involved in DNA recombination and repair, and proteins involved in carbon *trans*port and metabolism. Proteins of the TIGR00266 family and hypothetical proteins with unknown function were also detected.

In addition, we identified several proteins secreted by the oraCMU strain of which some were related to bacterial cell wall damage and the modulation of inflammation. Among the identified proteins, *N*-acetylmuramidase may be a specific candidate as an antibacterial substance. *N*-acetylmuramidase, also called lysozyme, has an enzymatic activity that cleaves the 1,4-linkage between *N*-acetylmuramic acid and *N*-acetylglucosamine in the peptidoglycan found in the bacterial cell wall [33]. Interestingly, the result of this study showed that this protein may cause lysis of *P. gingivalis* after binding to its cell wall, but does not affect *P. intermedia* and *F. nucleatum*.

*P. gingivalis*, as a major periodontopathogen, plays a role in the reduction of the secreted leukocyte protease inhibitor [34]. It has been suggested that the concentration of secreted leukocyte protease inhibitors, including the leukocyte elastase inhibitor (LEI) are likely to reduce periodontitis [34,35]. LEI, also known as a porcine serine protease inhibitor (serpin B1), belongs to the protein clade founded by ovalbumin [36]. On the other hand, MRS broth is known to contain abundant amounts of serpin B1 [37]. Sánchez et al. [38] reported that the same serpin is secreted by *L. rhamnosus* GG, and it is presumed to be a proteolytic product cleaved by *L. rhamnosus* GG protease. It is important to note that these are crude extracts that also include proteins derived from the MRS broth, meaning that the identified LEI may already be present in the broth. Interestingly, only the substance BLC of CFS in the oraCMU showed any antibacterial effect in this study. Furthermore, our previous study showed that oraCMU has the capability of inhibiting *F. nucleatum*-induced interleukin-6 and interleukin-8 production [20]. Therefore, our hypothesis is that the secretory proteins of oraCMU strains are likely to be involved in immunomodulatory processes.

In summary, our study examined the antibacterial properties of oraCMU on periodontopathogenic bacteria. Our results suggest that there are at least three different mechanisms by which oraCMU suppresses the growth of periodontopathogenic bacteria: an acidic pH, production of hydroxyl radicals, and the secretion of specific proteins with antimicrobial activity. Lactic acid, acetic acid and citric acid as organic acids, and oleic acid as fatty acids, were identified as potential antibacterial compounds. The H_2_O_2_-secretion ability of oraCMU under aerobic conditions was better than that under anaerobic conditions. Specific proteins, such as *N*-acetylmuramidase, secreted by the probiotic bacterium oraCMU was identified, which could contribute to its antimicrobial properties. Additionally, LEI may be responsible for the beneficial effects of oraCMU through their interaction with host cells. These secreted proteins could be involved in the probiotic effects exerted by oraCMU. Further studies are needed to evaluate the potential of the identified proteins.

## 4. Materials and Methods 

### 4.1. Bacterial Strains and Growth Conditions

*W. cibaria* CMU (oraCMU; Oradentics Inc., Seoul, South Korea), which has oral care probiotic properties, was used as a model microorganism [4,17,18,19,20]. *W. cibaria* was grown aerobically in MRS broth (Difco, Detroit, MI, USA) at 37 °C for 16 h. To prepare the CFS of bacterium, the cells were removed by centrifugation (4000× *g*, 20 m, 4 °C) and the supernatant was filter-sterilized (0.45 μm pore size; Millipore, Burlington, MA, USA). *P. gingivalis* KCTC 5352 and *F. nucleatum* KCTC 2488 were purchased from the Korean Culture Collection for Type Cultures (Daejun, South Korea). *P. intermedia* ATCC 25,611 was kindly provided by the Chonnam National University (Gwangju, Korea) *F. nucleatum* and *P. intermedia* were grown anaerobically in brain heart infusion broth (BHI broth, Difco) supplemented with 1% yeast extract (Difco), 0.1% cysteine (Sigma, St. Louis, MO, USA), 5 μg/mL hemin (Kisan Bio Co., Ltd., Seoul, South Korea), and 0.5 μg/mL menadione (Kisan Bio) at 37 °C for 48 h. *P. gingivalis* was grown anaerobically in tryptic soy broth (Kisan Bio) supplemented with 5 μg/mL hemin and 0.5 μg/mL menadione at 37 °C for 48 h.

### 4.2. Growth Inhibition of Periodontopathogenic Bacteria 

The CFS of probiotics was tested against *F. nucleatum*, *P. gingivalis*, and *P. intermedia* to determine its dose-dependent inhibitory activity, reflecting the activity of the organic acids, H_2_O_2_, or BLC, according to a previous method [39]. The inhibitory effects of organic acids on growth were determined after mixing CFS with proteinase K (0.1 mg/mL; Sigma) and catalase (0.05 mg/mL; Sigma). The hydrogen peroxide-dependent activity was evaluated using neutralized and proteinase K-treated CFS. BLC-dependent activity was analyzed with neutralized and catalase-treated CFS. CFS was 2-fold serial diluted and added to each well. Cultures of periodontopathogenic bacteria were adjusted to OD_600_ = 0.05 with each growth medium. The same quantity of CFS of each probiotic was inoculated in a 96-well plate containing periodontopathogenic bacteria. Each well was read at 600 nm with a microplate reader (VersaMax, Molecular devices, San Jose, CA, USA) after anaerobic incubation for 48 h at 37 °C.

### 4.3. SEM Analysis

SEM analysis was performed as described by Sorrentino et al. [40]. Briefly, bacterial culture grown for 16 h was centrifuged (4000× *g*, 20 min, 4 °C), pellet-washed 2 times with a phosphate buffer (PB), and fixed overnight at 4 °C in 2.5% glutaraldehyde in 0.1 M PB. After fixing, samples were rinsed 3 times with PB and 1% OsO_4_, dehydrated for 1 h, and rinsed 2 times (0.1 M PB and distilled water each) in a graded ethanol series (50%, 60%, 70%, 80%, 90%, and 100%) for 15 min each. For substitution, hexamethyldisilazane (Samchun Pure Chemical, Seoul, South Korea) was treated 2 times for 10 min. After drying it overnight, samples were sputter-coated with gold and observed through SEM (Hitachi, Model: S-4800, Tokyo, Japan) at an accelerating voltage of 1.0 kV.

### 4.4. H_2_O_2_ Determination 

In order to determine the kinetics of H_2_O_2_ production from the CFS under aerobic or anaerobic conditions, growth (OD_600_) of bacterial culture at 30 °C or 37 °C after 8, 16, and 24 h were measured simultaneously before centrifugation. CFS were then neutralized to pH 7.0, filter-sterilized (0.45 μm), and assayed for hydrogen peroxide according to the protocol (ab102500, Abcam, Cambridge, MA, USA) [4]. A 0.1-mL sample of supernatant placed in a microplate was read at 570 nm with a microplate reader. 

### 4.5. Analysis of Organic Acids by HPLC

Analysis of organic acids in the CFS was carried out according to the method described by Yeo and Liong [41] with some modifications. Briefly, filtered samples were injected (10 μL) in triplicate into a HPLC system (Shiseido, Tokyo, Japan) equipped with Prevail Organic Acid column (250 × 4.6-mm i.d.), maintained at 40 °C. Organic acids (oxalic, citric, succinic, lactic, and acetic acids) were quantified by using a PDA detector (Shiseido Co., Ltd., Tokyo, Japan) monitoring the absorbance at 214 nm. Under these selected chromatographic conditions, the chromatogram of the standard mixture of all organic acids was obtained. A degassed mobile phase (0.25 M KH_2_PO_4_ with pH = 2.4) was used at a flow rate of 0.4 mL/min. Standards (Sigma) were prepared in deionized filter-sterilized water.

### 4.6. Analysis of Fatty Acids by GC

The crude lipid fraction was converted to fatty acid methyl esters (FAME) through sodium methoxide catalysis [28]. The FAMEs were analyzed on SP-2560 columns (100 m × 0.25 mm × 0.20 µm) using an Agilent Model 6850 gas chromatograph equipped with an FID (Agilent Technologies, Little Falls, PA, USA). Operating conditions were as follows: injector, 225 °C; detector, 285 °C; initial, 100 °C (hold 4 min); ramp, 208 °C (3 °C/3 min); final, 244 °C (hold 15 min); carrier gas, helium; flow rate, 0.75 mL/min; linear velocity, 18 cm/s; and split ratio, 200:1. The injection volume was 10 μL. Supelco 37 Component FAME Mix (Sigma) was used as a standard.

### 4.7. Analysis of Secretory Proteins by MALDI-TOF MS

All chemicals used in this study were purchased from Sigma. To identify secretory proteins of oraCMU, CFS was mixed with 7 M urea, 2 M Thiourea containing 4% (*w*/*v*) 3-[(3-cholamidopropy) dimethyammonio]-1-propanesulfonate (CHAPS), 1% (*w*/*v*) dithiothreitol (DTT), 2% (*v*/*v*) pharmalyte, and 1 mM benzamidine for 1 h. After centrifugation at 15,000× *g* for 1 h at 15 °C, soluble fractions (200 μg) were separated on 2D gel electrophoresis and then silver-stained, as described by Oakley et al. [42]. Quantitative analysis of digitized images was performed using the PDQuest (version 7.0; Bio-Rad Laboratories, Hercules, CA, USA) software according to the manufacturer’s protocols. Protein spots were cut from the gel for protein identification by peptide mass fingerprinting, digested with trypsin (Promega, Madison, WI, USA), resuspended in α-cyano-4-hydroxycinnamic acid in 50% acetonitrile/0.1% trifluoroacetic acid, and analyzed by MALDI-TOF MS analysis (Microflex LRF 20, Bruker Daltonics, Billerica, MA, USA) as described by Fernandez et al. [43]. Spectra were collected at 300 shots per spectrum in the range of 600–3000 Da and calibrated with two-point internal calibration using trypsin auto-digestion peaks. The peak list was generated using Flex Analysis 3.0 (Bruker Daltonics). The data were analyzed with MASCOT software.

## Figures and Tables

**Table 1:** Quantitative analysis of organic and fatty acids in the CFS of oraCMU after 16 h incubation at 37 °C.

Acids	mg/mL	Acids	mg/mL	Acids	mg/mL
Oxalic acid	ND	Pentadecanoic acid (C15:0)	ND	*cis*-11-Eicosenoic acid (C20:1)	ND
Citric acid	0.88 ^a^	*cis*-10-Peptadecenoic acid (C15:1)	ND	*cis*-11,14-Eicosadienoic acid (C20:2)	ND
Succinic acid	ND ^b^	Palmitic acid (C16:0)	0.00	*cis*-11,14,17-Eicosatrienoic acid (C20:3n-3)	ND
Lactic acid	6.42	Palmitoleic acid (C16:1)	ND	*cis*-8,11,14-Eicosatrienoic acid (C20:3n-6)	ND
Acetic acid	2.78	Heptadecanoic acid (C17:0)	ND	Arachidonic acid (C20:4n-6)	ND
Butyric acid (C4:0)	ND	*cis*-10-Heptadecenoic acid (C17:1)	ND	*cis*-5,8,11,14,17-Eicosapentaenoic acid (C20:5n-3)	ND
Caproic acid (C6:0)	ND	Stearic acid (C18:0)	ND	Heneicosanoic acid (C21:0)	ND
Caprylic acid (C8:0)	ND	Elaidic acid (C18:1n-9, *trans*)	ND	Behenic acid (C22:0)	ND
Capric acid (C10:0)	ND	Oleic acid (C18:1n-9, *cis*)	0.2	Erucic acid (C22:1n-9)	ND
Undecanoic acid (C11:0)	ND	Linoleiaidic acid (C18:2n-6, *trans*)	ND	*cis*-13,16-Docosadienoic acid (C22:2)	ND
Lauric acid (C12:0)	ND	Linoleic acid (C18:2n-6, *cis*)	ND	*cis*-4,7,10,13,16,19-Docosahexaenoic acid (C22:6n-3)	ND
Tridecanoic acid (C13:0)	ND	α-Linolenic acid (C18:3n-3)	ND	Tricosanoic acid (C23:0)	ND
Myristic acid (C14:0)	ND	γ-Linolenic acid (C18:3n-6)	ND	Lignoceric acid (C24:0)	ND
Myristoleic acid (C14:1)	ND	Arachidic acid (C20:0)	ND	Nervonic acid (C24:1)	ND

^a^ Data expressed as the mean. ^b^ ND: not detected.

**Table 2:** Secreted proteins identified in the CFS of oraCMU.

Spot No. ^a^	Protein Description ^b^	Accession Number ^c^	MW (kDa)	PI	Protein Score CI (%) ^d^
1a–e	Type 1 glyceraldehyde-3-phosphate dehydrogenase	WP_010369259	35.756	5.03	100.00
2a–c	*N*-acetylmuramidase	WP_010373609	29.343	5.40	100.00
3	TIGR00266 family protein	WP_043941068	24.976	4.91	99.19
4a–f	Leukocyte elastase inhibitor	XP_003482167	42.658	6.13	100.00
5a–c	l-lactate dehydrogenase	WP_010372268	33.942	4.75	100.00
6a,b	Hypothetical protein	WP_043711225	23.526	5.60	99.99
		WP_063083480	23.318	5.04	100.00
7	Aldo/keto reductase	WP_063083131	36.627	5.08	100.00
8a,b	Single-stranded DNA-binding protein	WP_056973096	17.991	4.99	100.00
		WP_043709699	15.326	5.17	99.99
9a,b	NAD-dependent glyceraldehyde-3-phosphate dehydrogenase	KKU03010	41.843	5.54	100.00
10	*N*-acetylglucosamin-6-phosphate deacetylase	WP_0432710743	42.715	5.17	100.00
11	BMP family ABC *trans*porter substrate-binding protein	WP_060655478	39.514	9.18	100.00
12	Nucleoside-diphosphate kinase	WP_043710743	42.715	5.17	100.00
13	Glyceraldehyde 3-phosphate reductase	WP_010370341	36.629	5.00	100.00
14	16S rRNA (cytosine(1402)-*N*(4))-methyl*trans*ferase RsmH	WP_004044730	35.71	8.73	99.64
15	HTH-type *trans*criptional activator RhaR	WP_008787014	33.031	6.59	99.94
16	2,3-diphosphoglycerate-dependent phosphoglycerate mutase	WP_010371458	26.717	4.94	100.00
17	Phosphocarrier protein HPr	WP_010370017	9.191	4.57	99.19
18	PTS mannose *trans*porter subunit EIIAB	WP_043709678	35.417	4.97	100.00
19	Zinc-dependent alcohol dehydrogenase	XP_003482167	42.658	6.13	99.99

^a^ Spot numbers refer to the proteins labeled in Figure 4. ^b^ Protein description derived from the MASCOT software (www.matrixscience.com). ^c^ Information obtained from the NCBI database (www.ncbi.nih.gov). ^d^ CI, confidence interval.
